# A Distinct Faecal Microbiota and Metabolite Profile Linked to Bowel Habits in Patients with Irritable Bowel Syndrome

**DOI:** 10.3390/cells10061459

**Published:** 2021-06-10

**Authors:** Bani Ahluwalia, Cristina Iribarren, Maria K. Magnusson, Johanna Sundin, Egbert Clevers, Otto Savolainen, Alastair B. Ross, Hans Törnblom, Magnus Simrén, Lena Öhman

**Affiliations:** 1 Department of Microbiology and Immunology, Institute of Biomedicine, University of Gothenburg, 405 30 Gothenburg, Sweden; bani.ahluwalia@gu.se (B.A.); cristina.iribarren.gomez@gu.se (C.I.); maria.magnusson@microbio.gu.se (M.K.M.); johanna_sundin@hotmail.com (J.S.); 2 Calmino Group AB, Research and Development, 413 46 Gothenburg, Sweden; 3 Department of Molecular and Clinical Medicine, Institute of Medicine, University of Gothenburg, 413 45 Gothenburg, Sweden; eclevers@turner.nl (E.C.); hans.tornblom@gu.se (H.T.); magnus.simren@medicine.gu.se (M.S.); 4 GI Motility and Sensitivity Research Group, Translational Research Centre for Gastrointestinal Disorders (TARGID), KU Leuven, 3000 Leuven, Belgium; 5 Chalmers Mass Spectrometry Infrastructure, Department of Biology and Biological Engineering, Chalmers University of Technology, 412 96 Gothenburg, Sweden; otto.savolainen@chalmers.se (O.S.); Alastair.Ross@agresearch.co.nz (A.B.R.); 6 Proteins and Metabolites Team, AgResearch, Lincoln 7674, New Zealand; 7 Center for Functional Gastrointestinal and Motility Disorders, Division of Gastroenterology & Hepatology, School of Medicine, University of North Carolina at Chapel Hill, Chapel Hill, NC 27599, USA

**Keywords:** irritable bowel syndrome, pathophysiology, intestinal microenvironment, microbiota, microbial metabolites

## Abstract

Patients with irritable bowel syndrome (IBS) are suggested to have an altered intestinal microenvironment. We therefore aimed to determine the intestinal microenvironment profile, based on faecal microbiota and metabolites, and the potential link to symptoms in IBS patients. The faecal microbiota was evaluated by the GA-map^TM^ dysbiosis test, and tandem mass spectrometry (GC-MS/MS) was used for faecal metabolomic profiling in patients with IBS and healthy subjects. Symptom severity was assessed using the IBS Severity Scoring System and anxiety and depression were assessed using the Hospital Anxiety and Depression Scale. A principal component analysis based on faecal microbiota (*n* = 54) and metabolites (*n* = 155) showed a clear separation between IBS patients (*n* = 40) and healthy subjects (*n* = 18). Metabolites were the main driver of this separation. Additionally, the intestinal microenvironment profile differed between IBS patients with constipation (*n* = 15) and diarrhoea (*n* = 11), while no clustering was detected in subgroups of patients according to symptom severity or anxiety. Furthermore, ingenuity pathway analysis predicted amino acid metabolism and several cellular and molecular functions to be altered in IBS patients. Patients with IBS have a distinct faecal microbiota and metabolite profile linked to bowel habits. Intestinal microenvironment profiling, based on faecal microbiota and metabolites, may be considered as a future non-invasive diagnostic tool, alongside providing valuable insights into the pathophysiology of IBS.

## 1. Introduction

Irritable bowel syndrome (IBS) is a multifactorial disease involving a perturbed gut–brain interaction [1], visceral hypersensitivity [2], altered gastrointestinal (GI) motility [3], increased permeability, immune activation [4] and an altered gut microenvironment [5]. Currently, the diagnosis of IBS is based on symptom-based criteria, and in most cases, a limited number of tests are carried out to exclude other organic GI diseases [6]. However, reliable disease-specific biomarkers for IBS are still lacking [6].

The microbiota inhabiting the human GI tract coexist under a mutualistic relationship and benefit the host by participating in immunity, resistance to pathogen colonization and intestinal development [7,8]. The gut microbiota produces a diverse range of metabolites from the anaerobic fermentation of exogenous undigested dietary compounds. These small and diverse molecules reach the colon and are used as energy sources and substrates in metabolic or signalling pathways, as well as influencing host immune response [9,10], reflecting the hosts’ physiological status [11]. Metabolites may be regarded as a “fingerprint” of the functional interactions taking place between the host and microbiota [9]. Recent studies have described an altered composition of gut microbiota and metabolites in several diseases of the GI tract such as colorectal cancer and inflammatory bowel disease [11,12]. In addition, IBS has been linked to an unbalanced gut microbiota profile [13,14,15,16] and possibly also to an altered metabolite profile [17,18], with links to GI [17,19] and psychological symptoms [20], at least in subsets of patients. There are also contradictory reports of lower, as well as higher, concentrations of faecal short-chain fatty acids in IBS with diarrhoea (IBS-D) as compared to healthy subjects [21]. However, no clear differences have been depicted in the microbiome or metabolome profiles between IBS subtypes [22]. The published data within the field are still relatively limited and studies combining analyses of faecal microbiota and metabolites are rare and inconclusive [22,23,24,25,26]. Despite inconsistencies, the overall picture indicates that subsets of IBS patients have an altered intestinal microenvironment [27].

We hypothesized that the intestinal microenvironment differs between IBS patients and healthy subjects. Analyses of the joined faecal microbiota and metabolite composition may provide valuable insights into host–microbiota interactions and how these interactions can play a role in the pathophysiology of IBS. In this study, we aimed to determine the intestinal microenvironment, based on both faecal microbiota and metabolites, and its link to bowel habits, symptom severity and psychological symptoms in patients with IBS.

## 2. Materials and Methods

### 2.1. Study Cohort

This is a retrospective study analysing faecal samples collected from IBS patients and healthy subjects recruited as described previously [16,28]. Briefly, IBS patients (18–70 years old) meeting the Rome III criteria [29] were recruited from patients referred to the gastroenterology outpatient clinic at the Sahlgrenska University Hospital, Gothenburg, as well as through advertisement in local newspapers. Exclusion criteria were the presence of severe diseases such as malignancy, heart disease, kidney disease; neurological or psychiatric diseases; or a GI disease other than IBS that could explain their current symptoms (e.g., inflammatory bowel disease, coeliac disease). Patients on special diets such as the low FODMAP diet and low fibre diet were not included in the study. IBS patients were characterized as having IBS with constipation (IBS-C), diarrhoea (IBS-D) [29], mixed IBS (mixed loose and hard stools) (IBS-M) or unclassified IBS (IBS-U) by evaluation of bowel movements (number of stools per day) and stool consistency according to Bristol Stool Form (BSF) scale in a two-week stool diary. For analyses, the last two subtypes, IBS-M and IBS-U, were grouped together as IBS-nonCnonD [29].

All IBS patients completed validated self-report questionnaires assessing the severity of their IBS symptoms (IBS Severity Scoring System; IBS-SSS) [30], and anxiety and depression (Hospital Anxiety and Depression Scale; HADS) [31]. These questionnaires have validated cut-off levels used to categorize patients according to symptom severity and the presence or absence of anxiety or depression, respectively [30,32] (see Supplementary Material for more details). Healthy subjects (18–70 years old) with no current or prior history of GI diseases were included as controls. Chronic disorders, use of any immunosuppressive agents, antibiotics or any other medication during the 3 months prior to sample collection, and being on restrictive diets (i.e., vegan, glute-free and lactose-free diet), were reasons for exclusion from the healthy cohort. The use of other special diets was recorded as previously shown [16]. Pregnant or lactating women were not included. Additionally, all study subjects were requested to abstain from the intake of pre- and probiotics. All IBS patients and healthy subjects were given verbal and written information before giving their written consent to participate in the study. The study protocol was approved by the Regional Ethical Review Board in Gothenburg. All authors had access to the study data and have reviewed and approved the final manuscript.

### 2.2. Faecal Sample Collection

Faecal samples were collected by all study subjects at home. Study subjects were provided with faecal sample collection kits, including an empty tube and a sampling spatula. Samples were kept in a freezer at −20 °C until being transferred to the laboratory in a mini cooler bag with an ice pack. Samples were stored in the laboratory at −80 °C until analysis. All samples were analysed for microbiota and metabolomics profiles, respectively.

### 2.3. Faecal Microbial Analysis

Bacterial populations were detected at Genetic Analysis AS (Oslo, Norway) using the commercially available analysis, the GA-map™ dysbiosis test. The GA-map^TM^ test targets ≥ 300 bacteria belonging to different taxonomic levels by using a set of 54 highly specific DNA probes previously defined to discriminate between healthy subjects and patients with IBS in faecal samples. The protocol is described in detail elsewhere [33]. Briefly, this test includes faecal sample homogenization, mechanical bacterial cell disruption and automated total bacterial genomic DNA extraction with magnetic beads. Amplification of 16S rRNA (V3–V9 regions) is performed by polymerase chain reaction (PCR), followed by a single-nucleotide extension reaction. In this step, the complementary PCR amplicon hybridizes with a labelling probe, which is then extended with a labelled nucleotide. Subsequent addition of complementary probes coupled with magnetic beads hybridize to the labelled probes. This last hybridisation provides the signal from the nucleotide labelled-labelling probes that is acquired by the BioCode 1000A 128-Plex Analyzer (Applied BioCode, Santa Fe, CA, USA). This signal corresponds to the bacteria present in the sample, which are identified with the help of magnetic beads [33]. As a result, the GA-map^TM^ test generates a bacterial profile based on absolute faecal bacterial abundance, which is denoted as probe signal intensity [33].

### 2.4. Faecal Supernatant Preparation and Metabolite Analysis

Faecal supernatants extracted from faecal samples were used for metabolomics analysis. Faeces were mixed with 2 weight volumes of ice-cold PBS, followed by centrifugation for 10 min at 1600× *g*. The resulting supernatant was ultra-centrifuged at 35,000× *g* for 2 h, at 4 °C. The faecal supernatants were collected and stored at −80 °C until analysis. The metabolomic profile of faecal supernatant samples was analysed using gas chromatography coupled to a tandem mass spectrometer (GC-MS/MS) using the method of Savolainen et al. [34]. Briefly, samples were extracted with water:methanol (1:9 *v*/*v*) containing ten stable isotope labelled internal standards [34], followed by drying and derivatization by using oxymation and silylation. The derivatised extracts were injected into a GC-MS/MS system (Shimadzu GCMS TQ-8030 system, Shimadzu Europa GmbH, Duisberg, Germany) and GC-MS scan data (between *m*/*z* 50–750) were analysed for targeted peak detection. Metabolites present in the Swedish Metabolomics Centre library were screened against the scan data using a Matlab script (Mathworks, Natick, MA, USA) and database developed at the Swedish Metabolomics Centre (Umeå, Sweden). This uses the retention index, diagnostic ion and spectral matching to score and identify likely metabolites. Peaks were visually inspected to ensure they were correct. Data were normalized based on the internal standard peak intensities [35].

### 2.5. Statistical Analyses

Univariate statistical analysis was performed using the SPSS Statistical Package (IBM Corp. Released 2017. IBM SPSS Statistics for Windows, Version 25.0. Alrmonk, NY, USA: IBM Corp.). Categorical variables were compared with the χ^2^ test, whereas continuous variables were compared using either the independent samples and paired samples *t*-test or the corresponding non-parametric Mann–Whitney U test and Wilcoxon signed rank test, based on the normality of distribution determined by histograms and Kolmogorov–Smirnov statistic tests. The data in text, figures and tables are shown, corresponding to the distribution, as mean ±SD or median (interquartile range), respectively. *p* values < 0.05 were considered statistically significant.

Multivariate analyses were applied to the combined dataset containing faecal microbiota and metabolites data. Principal component analysis (PCA) was conducted using the *prcomp*-function with z-score scaling, and visualized using the *pca3d*-package in R (version 3.6.2, Vienna, Austria) [36]. The multivariate group means were represented as centroids. To test the statistical significance of comparisons between the two groups (e.g., IBS vs. healthy), i.e., whether the groups differed in their mean scores on the two principal components, we iteratively randomized the group labels to simulate a null-distribution of centroid differences, and ranked the actual value within this null-distribution. *p* values < 0.05 were interpreted as significantly different multivariate group means. Orthogonal partial least squares-discriminant analysis (OPLS-DA) was implemented to investigate if the combined dataset, containing faecal microbiota and metabolites profiles (X variables), could discriminate IBS patients from healthy subjects (Y variables). All discriminant analyses were performed using SIMCA^®^ software (version 15.0.2, MKS Umetrics AB, Umeå, Sweden). The quality of OPLS-DA was determined based on the parameters R^2^, i.e., the goodness of fit of the model (values of ≥0.5 define good discrimination, where R^2^ = 1 is the best possible fit), and Q^2^, i.e., the goodness of prediction of the model (values of ≥0.4 are considered good) [37]. The reliability of the models was confirmed using analysis of variance testing of cross-validated predictive residuals (CV-ANOVA), where a *p* value < 0.05 indicated significantly different residuals of the compared groups. Student’s *t*-test was used to verify the robustness of the selected variables from the simplified OPLS-DA model.

Volcano scatter plots were used for the identification of differentiating faecal microbiota and metabolites for which the fold change for each metabolite and bacterial taxon between IBS patients and healthy subjects was calculated, i.e., (metabolite A in IBS/metabolite A in healthy) and (bacterial taxon A in IBS/bacterial taxon A in healthy). Significance (–log_10_ (*p* value < 0.05), Student’s *t*-test) versus log_2_ (mean fold change) [38] were plotted, and only most relevant variables were labelled. Visualization was performed in R using the *ggplot2* and *ggrepel* packages. Further, Cleveland plot analysis was performed in R using the *ggplot2*, *dplyr* and *tidyr* packages.

In addition, the least absolute selection and shrinkage operator (LASSO) method with regularization was used to extract a set of most relevant discriminative variables amongst microbial taxa and metabolites to help distinguish IBS patients from healthy subjects and further facilitate the interpretability of our findings by simplifying our model. LASSO was performed using the *caret* [39] and *glmnet* [40] packages in R and is described in detail in the Supplementary material.

The ingenuity pathway analysis (IPA, version 2.3) (Qiagen Inc., https://www.qiagenbio-informatics.com/products/ingenuitypathway-analysis, trial version accessed on 18 March 2020) [41] was used to identify biological networks associated with mean fold change values (IBS patients/healthy subject) for each of the analysed faecal metabolites. A *p* value < 0.05 was set as the cut-off and activation z-scores were calculated and used to predict increased (z-score > 2.0) or decreased (z-score < −2.0) biological functions that could explain the differences on the metabolite dataset.

## 3. Results

### 3.1. Characteristics of the Study Cohort

The study cohort consisted of 40 IBS patients and 18 healthy subjects. The demographics and clinical characteristics of the study subjects are shown in Table 1. Females and males were unequally distributed between the study groups, and healthy subjects were younger than IBS patients (*p* < 0.001). All IBS subtypes (IBS with diarrhoea or IBS-D, IBS with constipation or IBS-C; and IBS-nonCnonD or combined mixed-type IBS and IBS unclassified) were represented in the study population. The majority of patients presented with moderate to severe IBS symptoms (IBS-Severity Scoring System; IBS-SSS) and had higher Hospital Anxiety and Depression Scale (HADS) total scores than healthy subjects. Likewise, a higher proportion of individuals with anxiety was found among the IBS patients compared to healthy subjects, whereas the proportion of individuals with depression did not differ between the two groups.

### 3.2. The Intestinal Microenvironment Differs between IBS Patients and Healthy Subjects

**Figure 1:**
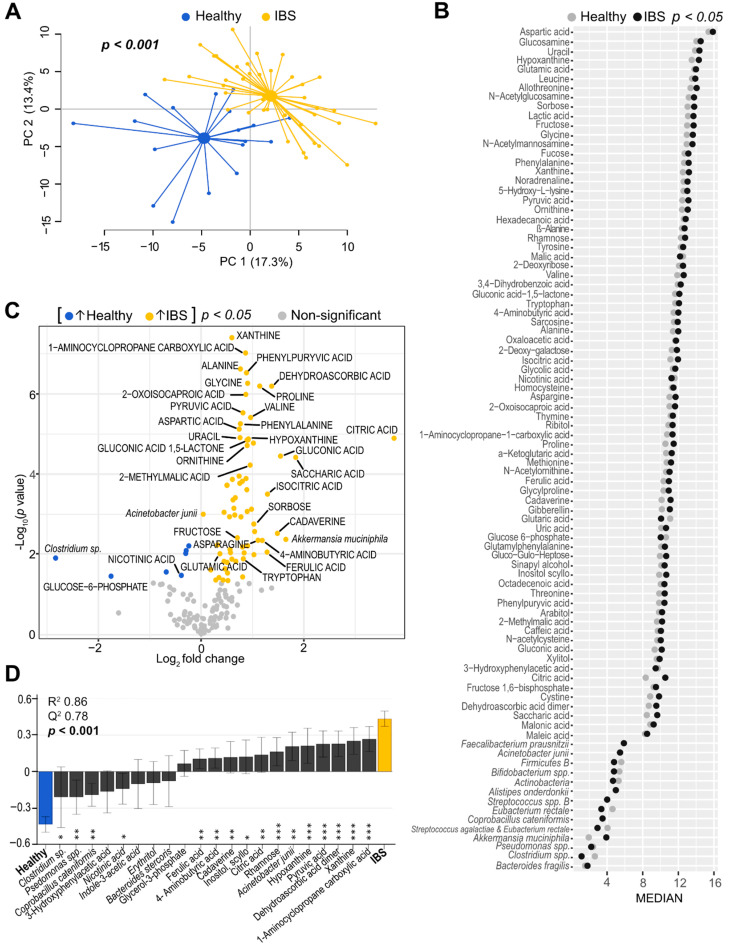
Faecal microbiota and metabolite profiles of IBS patients and healthy subjects. (**A**) Principal component analysis (PCA) score scatter plot based on faecal bacteria and metabolites showing IBS patients (*n* = 40, yellow dots) and healthy subjects (*n* = 18, blue dots). The *p* value depicts statistical significance of the distance between centroids (scores average) of the groups. (**B**) Cleveland dot plot depicting the differentiating microbiota and metabolite variables (*p* < 0.05) between IBS patients and healthy subjects. The horizontal axis represents bacterial probe intensity (*n* = 14) and metabolite signal intensity (*n* = 76) values and the vertical axis shows the variable IDs. Log-transformed median values for each X-variable are depicted for the IBS group (black dots) and healthy group (grey dots), respectively. Mann–Whitney U test was performed to evaluate differences between the groups. (**C**) Volcano plot illustrating mean fold change vs. statistical significance based on 54 bacteria (probe intensity) and 155 metabolites (signal intensity) in IBS patients vs. healthy subjects (IBS/H). Each dot corresponds to a bacterial probe or metabolite signal. Variables increased in IBS are coloured in yellow (*n* = 70), while decreased variables compared to healthy subjects are coloured in blue (*n* = 7). Mean fold change is shown as log_2_. *p* values are shown as –log_10_; *p* value < 0.05 are coloured; non-significant *p* values are shown in grey. Student’s *t*-test was used to identify differences between the groups. For visual clarity, only some bacteria and metabolites with log_2_ (IBS/H) < −1 and >+1 and *p* values < 0.05 are labelled. (**D**) Orthogonal partial least squares-discriminant analysis (OPLS-DA) loading column plot based on the discriminatory bacterial taxa (*n* = 5) and metabolites (*n* = 16), selected after the least absolute selection and shrinkage operator (LASSO) method with regularization, between IBS patients and healthy subjects. The R^2^ value indicates goodness of the fit; the Q^2^ value expresses prediction ability. The *p* value depicts statistical significance based on the cross-validated residuals (CV-ANOVA) test. Error bars indicate 95% confidence intervals. Asterisks represent significant *p*-values: * < 0.05; ** < 0.01; *** < 0.001.

First, we investigated whether the intestinal microenvironment differed between IBS patients and healthy subjects. A principal component analysis (PCA) based on the total number of identified faecal microbiota (*n* = 54 variables) and metabolites (*n* = 155 variables) showed a clear separation between IBS patients and healthy subjects, with a large distance between the centroids (scores averages) of the two groups (*p* < 0.001) (Figure 1A). This separation was not seen to be influenced by age and gender differences (Figure S1A,B). An orthogonal partial least squares-discriminant analysis (OPLS-DA), based on the total number of identified variables, further supported the differentiation seen between IBS patients and healthy subjects (Table 2). As visualized by Cleveland plots, the majority of the differentiating variables were found in higher abundance in IBS patients (bacterial taxa; *n* = 3 and metabolites; *n* = 67) compared to healthy subjects (bacterial taxa; *n* = 5 and metabolites; *n* = 2) (Figure 1B, Figure S2). Furthermore, a volcano plot showed that a large number of metabolites such as citric acid, alanine, glycine, tryptophan, xanthine, hypoxanthine and pyruvic acid along with *Acinetobacter junii* were higher in IBS patients as compared to healthy subjects, whereas only a few variables such as *Clostridium* sp., glucose-6-phosphate and nicotinic acid were found in lower abundance (Figure 1C). In addition, LASSO variable selection identified a total of 21 variables (bacterial taxa; *n* = 5 and metabolites; *n* = 16), providing an even more robust model (accuracy of 90.9%) for distinction of IBS patients from healthy subjects. The variables identified by the LASSO model as being most important for the separation between IBS patients and healthy subjects were depicted by an OPLS-DA (Figure 1D).

### 3.3. The Intestinal Microenvironment Differs between IBS Subgroups

**Figure 2:**
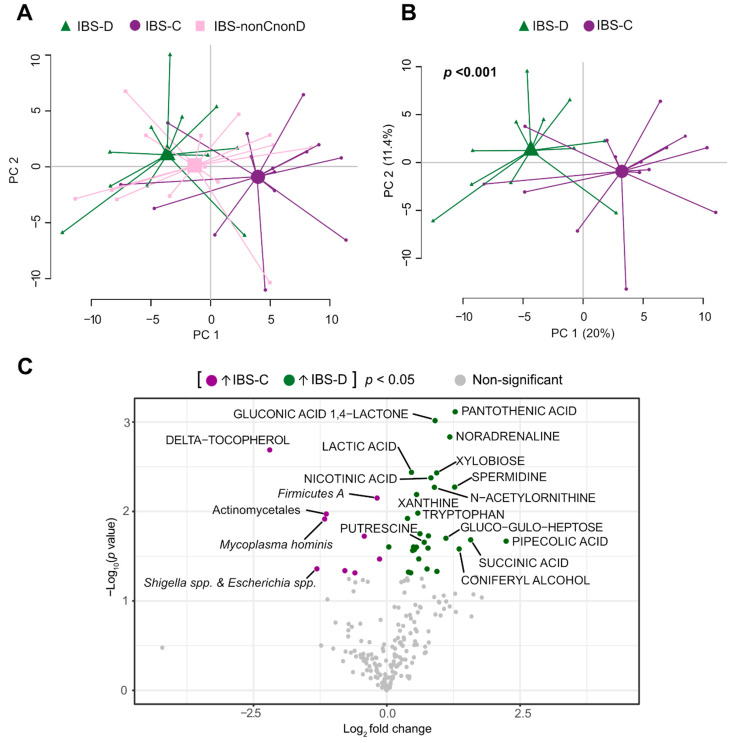
Faecal microbiota and metabolite profiles of IBS patients based on the predominant bowel habit. Principal component analysis (PCA) score scatter plot based on faecal bacteria and metabolites showing (**A**) IBS-D (*n* = 11, green triangles), IBS-C (*n* = 15, purple circles) and IBS-nonCnonD (*n* = 14, pink squares) patients and (**B**) patients with the predominant bowel habit, IBS-D and IBS-C. The *p* value depicts the statistical significance of the distance between the centroids (scores averages) of the groups. (**C**) Volcano plot illustrating statistical significance versus mean fold change of 54 bacteria (probe intensity) and 155 metabolites (signal intensity) between IBS-D patients respect to IBS-C patients (IBS-D/IBS-C). Each dot corresponds to a bacteria probe or metabolite signal. Variables increased in IBS-D are coloured in green (*n* = 29), while decreased variables compared to IBS-C are coloured in purple (*n* = 9). Mean fold change is shown as log_2_. *p* values are shown as –log_10_; *p* values < 0.05 are coloured; non-significant *p* values are shown in grey. Student’s *t*-test was used to identify differences between the groups. Not all variables were labelled, only some bacteria and metabolites with log_2_ (IBS/H) < −1 and >+1 and *p* values < 0.05.

We further explored whether the intestinal microenvironment composition differed between subgroups of IBS patients classified according to the predominant bowel habit, symptom severity and anxiety. A PCA based on the total number of identified faecal microbiota (*n* = 54) and metabolites (*n* = 155) showed that IBS-nonCnonD (*n* = 14) overlapped with IBS-C (*n* = 15) and IBS-D (*n* = 11) (Figure 2A), whereas patients with IBS-D clustered separately from IBS-C with significant distance between their centroids (*p* < 0.001) (Figure 2B). The separation between the predominant bowel habit subtypes was supported by an OPLS-DA based on the total number of measured variables (Table 2). Furthermore, a volcano plot identified the variables, mostly metabolites, which contributed to the separation between IBS-D (bacterial taxa; *n* = 2 and metabolites; *n* = 27) and IBS-C patients (bacterial taxa; *n* = 4 and metabolites; *n* = 5) (Figure 2C). IBS-D patients were defined by higher levels of numerous metabolites, whereas IBS-C patients were associated with higher levels of δ-tocopherol, *Mycoplasma hominins*, Firmicutes A, actinomycetales and *Shigella* sp. and *Escherichia* spp. (Figure 2C).

**Figure 3:**
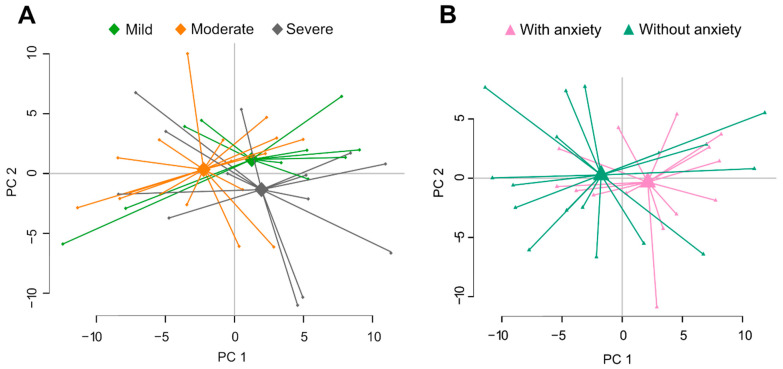
Faecal microbiota and metabolite profiles of IBS patients classified according to symptoms. (**A**) Principal component analysis (PCA) score scatter plot based on faecal bacteria and metabolites showing patients with mild (*n* = 15, light green), moderate (*n* = 18, orange) and severe (*n* = 7, grey) IBS symptoms (IBS-SSS). (**B**) PCA score scatter plot based on faecal bacteria and metabolites showing IBS patients with (*n* = 17, pink) and without anxiety (*n* = 14, teal) (based on validated cut-off levels on HAD-A, ≥8).

In contrast, the intestinal microenvironment profile did not differ between patients with mild (*n* = 10), moderate (*n* = 17) and severe IBS symptoms (*n* = 13) (Figure 3A, Table 2). Additionally, no clustering, based on the faecal microbiota and metabolites, was seen between IBS patients with and without anxiety (cut-off HADS anxiety score ≥ 8) (Figure 3B, Table 2). Too few patients met the criteria for borderline or clinically significant depression to allow exploration of the relationship with intestinal microenvironment profile.

### 3.4. Altered Amino Acid Metabolism and Cell Signaling-Related Pathways in IBS Patients

**Figure 4:**
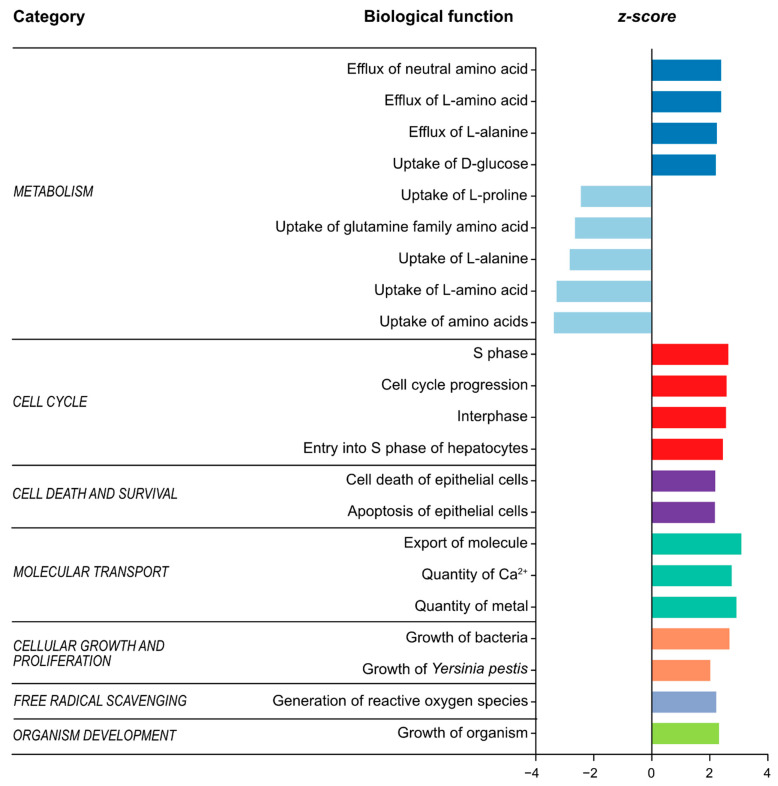
Significantly enriched biological functions predicted to be associated with changes in metabolites in patients with IBS compared to healthy subjects. The bar plot represents 22 biological functions predicted to be altered in IBS patients with significant activation Z-score > 2 or < −2 identified by IPA core analysis. Z-scores > 2 or < −2 indicate predicted decrease or increase, respectively. The analysis used a total of 35 metabolites (*p* value < 0.05). Each bar denotes a different biological function where each colour corresponds to a simplified category.

Finally, IPA “core analysis” was performed to explore if the distinct metabolite profile seen in IBS patients could be predicted to have an effect on biological functions. In all, from 500 total biochemical pathways identified with various biological function annotations, 22 biological functions were predicted to be differently regulated in IBS patients as compared to healthy subjects (Figure 4, Table S1). As identified by IPA, several functions related to metabolism, in particular amino acid metabolism, were predicted to be altered in IBS patients, based on upregulation in efflux of L-alanine along with downregulation in the uptake of L-proline and L-alanine, among others. Furthermore, functions such as the growth of bacteria and apoptosis of epithelial cells, included in the categories of proliferation of bacteria and cell death and survival, were predicted to be upregulated in IBS patients. Moreover, the biological functions of cell cycle, cellular growth and molecular transport were predicted to be upregulated in IBS, as seen in Figure 4 and Table S1.

## 4. Discussion

In this study, we demonstrated that IBS patients can be differentiated from healthy subjects based on the intestinal microenvironment, comprising the combined faecal microbiota and metabolite profiles. Additionally, we showed that IBS patients classified according to their predominant bowel habits, but not severity of IBS symptoms or presence/absence of anxiety, can be distinguished on the basis of the intestinal microenvironment. Further analysis suggested that altered amino acid metabolism and certain cell signalling pathways may be involved in the pathophysiology of IBS.

During the last decade, numerous studies have addressed the role of gut microbiota in IBS pathophysiology [13,14,15,16]. More recently, reports focusing on intestinal metabolites [17,18], alongside studies integrating the gut microbiome and metabolome [22,24,25], as well as the host epigenome and transcriptome [26], have explored host–microbiota interactions and the link to GI symptoms in IBS patients. Our current study demonstrates that a combined analysis of faecal microbiota and metabolite profiles separates a mixed group of IBS patients from healthy subjects. This is in agreement with a report by Shankar et al., who previously described this joint classifier to facilitate diagnosis of IBS and differentiate paediatric IBS-D patients from age-matched controls [24]. Furthermore, our study demonstrated that the combined microbiota and metabolite profiling model differentiated between IBS-D and IBS-C patients. This finding is in line with the work by Mars et al., reporting IBS subtype-specific and symptom-related variation in microbial composition and function [26]. Nevertheless, to emphasize the complexity of these analyses and their interpretation, there is also a report demonstrating a lack of differences between IBS subtypes based on the individual microbiota or metabolite profiles [22].

In this study, however, we did not detect differences in the intestinal microenvironment profile between IBS subgroups based on IBS symptom severity. Interestingly, our group previously demonstrated faecal bacteria to be associated with bowel habit-based IBS subtyping, whereas mucosa-associated bacteria were linked to IBS symptom severity [16]. It may therefore be suggested that the link between the intestinal microenvironment profile and bowel habits but not symptom severity in this study was due to the sampling site. Faecal samples, although not fully representative of the entire GI tract, are widely used in studies to reflect the intestinal microenvironment since they are non-invasive and easy to collect, compared to mucosal biopsies. Moreover, the analysis techniques used in this study to determine the microenvironment profile (GA-map^TM^ test and GC-MS/MS) are both relatively easier and quick to perform. This facilitates their use in a clinical setup in comparison to other explorative techniques, e.g., 16S rRNA gene sequencing and LC-MS/MS. The bacterial profiles generated by the commercially available GA-map^TM^ test based on faecal bacterial absolute abundance have the additional advantage of being closely associated with gastrointestinal disorders. Thus, the combination of the accessibility of faecal samples, and these two quick and reliable analyses may constitute an asset for development of non-invasive diagnostic tools.

Altered production or availability of metabolites such as amino acids, short-chain fatty acids, vitamins, bile salts, lipids and organic acids, associated with microbial metabolism or host-microbial co-metabolism, could result in alteration of pathways involved in host physiology [9]. Our study depicts a distinct IBS gut microenvironment characterized by increased amino acid metabolism seen as increase in alanine, glycine and proline, and amino acid metabolism intermediates such as phenylpyruvic acid and pyruvic acid. This may suggest higher epithelial cell turnover processes related to low-grade inflammation such as collagen degradation [42], potential malabsorption of amino acids [23], an increase in protein catabolism by the gut microbiota [25] or even increased efflux of amino acids in IBS patients. Additionally, increased levels of molecules involved in the Krebs cycle such as α-ketoglutaric acid, citric acid and pyruvic acid, but also hypoxanthine and xanthine, which are metabolic forms of purines, known to promote intestinal barrier development [43], were recorded in patients with IBS. This may indicate an altered energy metabolism and proliferation or degradation of bacterial and/or epithelial cells [23,44] in patients with IBS. Hypoxanthine is reported to promote intestinal barrier function and may, as a substrate for oxidase activity, play a role in IBS pathophysiology [43,45]. Indeed, IBS patients have been reported to display altered hypoxanthine levels, although results are inconclusive [18,26]. Still, the as yet scarce and somewhat inconsistent data of hypoxanthine in IBS highlight the complexity of this metabolic pathway, but also suggest that it may be of importance for underlying disease mechanisms. Moreover, our LASSO-extracted variables provided a refined discriminatory model, further implicating the importance of certain metabolic pathways such as the amino acid and purine metabolism. Altogether, our findings suggest that IBS patients may present alterations in their intestinal metabolic function and activity that could play an important role in the pathophysiology of IBS.

Growing evidence further indicates that IBS pathogenesis involves a dysregulated and bidirectional gut-brain interaction where microbiota and microbial metabolites influence neuro-immune crosstalk [1,20,46], and in return, the central nervous system influences the intestinal microenvironment [1]. A recent study demonstrated altered profiles of urine metabolites and faecal bacteria in IBS, and with specific correlations with anxiety and depression [20]. Even though the intestinal microenvironment profile defined in this study could not discriminate patients based on IBS severity or psychological symptoms, the detection of increased abundance of molecules related to neuro-immune signalling, such as tryptophan, ornithine, glycine and glutamic acid, may support an underlying perturbed gut-brain interaction in patients with IBS. Tryptophan, an intermediate metabolite in serotonin metabolism, has been suggested to be essential for the proliferation and effector functions of T cells [47] and the enhancement of serotonin production. Serotonin may regulate the activity of innate immune cells through interaction with receptors in mast cells, dendritic cells and macrophages/monocytes. Furthermore, microbial tryptophan catabolites are known to activate gut immune response via the aryl hydrocarbon receptor [48], potentially strengthening the intestinal barrier integrity [49]. Thus, an altered tryptophan metabolism may contribute to the aberrant host–microbial crosstalk associated with IBS patients. In addition, we also recorded increased levels of ornithine and its precursor glutamic acid in IBS patients. Both molecules are known to participate in the synthesis of polyamines as putrescine, spermidine and spermine, with a suggested importance in lymphocyte activation and development [50]. Moreover, tryptophan, glycine and glutamate which are metabolites involved in amino acid metabolism, have also been associated with IBS-specific brain changes in a recent study integrating functional neuroimaging and faecal metabolites [51]. All in all, these findings support the importance of metabolites in the aberrant brain–gut interaction implicated in the pathophysiology of IBS, warranting further investigations.

The IPA core analysis was carried out to evaluate the direction of the change in the metabolomic profile and predict effects on biological functions in IBS patients compared to healthy subjects. Interestingly, IPA further predicted the uptake of various amino acids to be altered in IBS patients, similarly to studies proposing differences in the ability of IBS patients to metabolize dietary substrates [23]. Additionally, the altered amino acid uptake may be associated with IBS-specific dietary habits suggested to be characterized by an increased protein and dietary fibre intake [52], although studies are inconclusive [53]. Additionally, apoptosis and cell death of epithelial cells were predicted to be increased in patients with IBS, supporting the notion of an impaired epithelial barrier in IBS pathophysiology [6]. It is also important to point out that the interactions between the intestinal microenvironment and the host are complex and multidimensional. Even though our results predict changes in several biological functions potentially associated with IBS disease pathophysiology, further metaproteomic analyses would provide supplementary functional information which would enhance the biological meaning of such distinct intestinal microenvironment. In line with this, a recent study identified peptides from potentially pathogenic *Brachyspira* in a subset of patients with IBS using metaproteomics, as well as described the immune response associated with this infection [54].

Additionally, our results suggest a relationship between bowel habits and the intestinal microenvironment, where a potential shift in microbial function rather than the microbial composition may be of importance and reflect an altered faecal metabolite profile in IBS subtypes. The separation between IBS-D and IBS-C patients was primarily attributed to higher levels of succinic acid, nicotinic acid and xanthine, which are involved in energy metabolism-related pathways. Similar to findings from Lee et al. [18], higher levels of tryptophan were also depicted in IBS-D patients. Tryptophan, as described above, is an intermediate metabolite in serotonin metabolism. Serotonin is known to promote motility, secretion and visceral hypersensitivity in IBS patients with diarrhoea [18,42,55] and, hence, drug development has to a large extent focused on 5-hydroxytryptamine (5-HT) receptor antagonist for symptom management [55]. Moreover, altered levels of tryptophan and putrescine observed in IBS-D could be implicated in symptom generation of abdominal pain and discomfort [56]. Overall, the distinct intestinal microenvironment associated with IBS-D patients described in our study is consistent with the current available literature [21,25,26].

While metabolites had greater influence when discriminating between IBS patients and healthy subjects, a few bacterial taxa were identified to be linked to health whereas others were associated with the IBS-specific profile. For instance, IBS patients had lower abundance of *Clostridium* sp. levels, suggested to be relevant in gut health [57]. A previous study has also associated members of Clostridiales including *Clostridium* sp., with GI sensorimotor function in healthy controls, but not in IBS patients [58]. These differences have been suggested to contribute to the altered brain-gut-microbe interaction and pain perception in patients with IBS [58]. Moreover, a higher abundance of Firmicutes, *Escherichia* spp. and *Shigella* sp. and *Mycoplasma homini* was a distinct feature of IBS-C patients in our study. These bacterial taxa have previously been associated with IBS, however not to any specific IBS subtype [14,59,60]. Altogether these results suggest that these different taxa may be associated with the predominant bowel habits in IBS patients.

Previous reports evaluating the effects of faecal microbiota transplantation (FMT) from healthy subjects, described that FMT normalized either microbiota [61] or microbiota together with short-chain fatty acids [62] in IBS-D patients and improved IBS symptoms. Altogether, these studies highlight differences in the intestinal microenvironment of IBS and further support the necessity to account for the role of both microbiota and metabolites when investigating new microbiota-based treatments for IBS management.

Although presenting promising results, this study has its limitations and weaknesses. First of all, human microbiome studies have raised concerns regarding the contribution of age and gender in the human gut microbiota [63]. Nevertheless, in spite of this retrospective study with limited study size was not matched for age and gender, this did not influence our findings. Moreover, dietary habits which can affect both the gut microbiota and metabolite composition [64] were not taken into account. Although this study collected only single time-point samples, longitudinal sampling has been shown to overcome heterogeneity and improve accuracy of results [26], and it would be a beneficial add on for future validation studies. Further, correction for stool water content or even the addition of a second healthy control group with an accelerated transit triggered by laxatives would have been valuable to support the effect of bowel habit on the intestinal microenvironment, as depicted in our study. Despite the limitations of the selected heterogeneous IBS cohort, we could demonstrate that a mixed IBS cohort could be differentiated from healthy subjects based on their intestinal microenvironment. Altogether, our study highlights the importance of the combined microbiota and metabolite profile in improving the ability to separate IBS patients and healthy subjects, and our refined model based on the LASSO selected variables provides possibilities for future explorative studies.

In conclusion, IBS appears to be associated with a shift in microbial function rather than microbial composition, with bowel habits also being reflected in the intestinal microenvironment profile of IBS patients. Accordingly, intestinal microenvironment profiling through measurement of specific faecal microbes and metabolites may be considered as a future non-invasive diagnostic tool alongside providing valuable insights into the pathophysiology of IBS.

## Figures and Tables

**Table 1:** Demographics and clinical characteristics of the study cohort.

Baseline Characteristics	IBS (*n* = 40)	Healthy (*n* = 18)	*p* Value
Sex (F:M)	31:9	9:9	0.04
Age, years ^†^	52 (24–70)	26 (19–54)	<0.001
IBS subtypes IBS-C:IBS-D:IBS-nonCnonD	15:11:14	N/A	
IBS-SSS ^‡^	236 (174–327)	11 (0–20)	<0.001
Mild:moderate:severe ^§^	10:17:13	N/A	
HADS total score ^¶^	9.9 ± 5.9	6.8 ± 3.9	<0.05
Anxiety:no anxiety ^φ^	14:17	3:15	0.04
Depression:no depression ^φ^	3:28	0:18	0.17

Note: Abbreviations: N/A, not applicable; IBS-C, IBS with constipation; IBS-D, IBS with diarrhoea; IBS-nonCnonD, mixed-type IBS and unclassified IBS; IBS-SSS, IBS Severity Scoring System; HADS, Hospital Anxiety and Depression Scale. ^†^ Data shown as median (min–max). ^‡^ Data shown as median (interquartile range). ^§^ Based on IBS-SSS. ^¶^ Data shown as mean ±SD. ^φ^ Based on HADS.

**Table 2:** Parameter values for orthogonal partial least squares-discriminant analysis (OPLS-DA) models.

OPLS-DA Model	R^2^	Q^2^	*p* Value
IBS vs. Healthy	0.96	0.79	<0.001
IBS vs. Healthy (LASSO) ^†^	0.86	0.78	<0.001
IBS-C vs. IBS-D	0.99	0.56	0.04
IBS-C vs. IBS-nonCnonD	0.46	0.14	0.13
IBS-D vs. IBS-nonCnonD ^‡^	0.75	−0.26	>0.99
IBS mild vs. IBS moderate ^§^	0.38	−0.005	>0.99
IBS mild vs. IBS severe ^‡,^^§^	0.84	−0.02	>0.99
IBS moderate vs. severe ^§^	0.42	0.05	0.52
IBS with anxiety vs. IBS without anxiety ^¶^	0.72	0.12	0.51

Note: All OPLS-DA models are based on the intestinal microenvironment profile, including 54 microbial taxa and 155 metabolites analysed, unless specified. R^2^ value indicates goodness of the fit of the model; Q^2^ value describes the prediction ability of the model. The *p* value depicts statistical significance based on ANOVA of the cross-validated residuals (CV-ANOVA). Abbreviations: IBS-C, IBS with constipation; IBS-D, IBS with diarrhoea; IBS-nonCnonD, mixed-type IBS and unclassified IBS. ^†^ IBS vs. healthy (LASSO) model based on 21 selected variables (*n* = 5 bacterial taxa and *n* = 16 metabolites). ^‡^ OPLS-DA model calculated by adding two first forced components. ^§^ Based on IBS-SSS, IBS Severity Scoring System. ^¶^ Based on Hospital Anxiety and Depression Scale.

## Data Availability

The data presented in this study are available on request from the corresponding author.

## Supplementary Materials

The following are available online at https://www.mdpi.com/article/10.3390/cells10061459/s1, Supplementary Methods: Symptom Assessment Questionnaires; and Selection of variables from the intestinal microenvironment profile, Figure S1: Faecal microbiota and metabolite profiles of IBS patients and healthy subjects, Figure S2: Cleveland dot plot representing the distinct combined microbiota and metabolite profile in IBS patients and healthy subjects, Table S1: Significantly enriched functions predicted by Ingenuity Pathway Analysis (IPA) “Core analysis”.
