# Supplementary material for: A Distinct Faecal Microbiota and Metabolite Profile Linked to Bowel Habits in Patients with Irritable Bowel Syndrome
Source: Cells. 2021 Jun 10;10(6):1459. doi: 10.3390/cells10061459 (PMC8230381; doi:10.3390/cells10061459)
Supplement: Supplementary file 1 [file cells-10-01459-s001.zip › cells-1241338-supplementary.pdf]

SUPPLEMENTARY MATERIAL

SUPPLEMENTARY METHODS

FIGURE S1 Faecal microbiota and metabolite profiles of IBS patients and healthy subjects. (A) Identified by gender. (B) **I**dentified by group of age.

FIGURE S2 Cleveland dot plot representing the distinct combined microbiota and metabolite profile in IBS patients and healthy subjects.

TABLE S1 Significantly enriched functions predicted by Ingenuity Pathway Analysis (IPA) “Core analysis”.

SUPPLEMENTARY METHODS

Symptom Assessment Questionnaires

Bristol Stool Form (BSF) scale

The BSF scale [1] was used to record stool frequency (number of stools per day), and mean stool consistency on a 7-point scale. The BSF scale was also used to determine the IBS subgroups, that is, IBS with constipation (IBS-C), IBS with diarrhoea (IBS-D), or mixed IBS (mixed bowel habits) (IBS-M) or unsubtyped IBS (IBS-U), where the two latter groups were combined into one group (IBS-nonCnonD).

IBS-Symptom Severity Scoring (IBS-SSS) System

The IBS-SSS was used to assess the severity of IBS symptoms. This questionnaire uses visual analogue scales (VAS) (0-100mm); the overall score is calculated from five items: pain severity, pain frequency, bloating severity, bowel habit dissatisfaction and influence of IBS on life in general or life interference, with a range of 0 to 500. This questionnaire allows the classification of patients into the following severity subgroups: mild (≤174 points), moderate (175 – 300), severe (>300) [2].

Hospital Anxiety and Depression Scale (HADS)

The HADS is a 14-item questionnaire used to measure the severity of anxiety and depression on 2 subscales with 7 items each. Each item is scored using a 4-point Likert scale, between 0 and 3, with higher scores indicating more severe symptoms. Total score range per subscale goes from 0 to 21 points and comprises three different subgroups [2,3]. The HAD subscale classifies patients without reported anxiety or depression (HAD score <8); borderline anxiety or depression (HAD score 8 - 10) or clinically significant anxiety or depression (HAD score ≥11).

In this study a HAD score ≥8 was considered as clinically significant anxiety or depression, while a HAD score <8 was considered as absence of anxiety or depression.

Selection of variables from the intestinal microenvironment profile

To simplify the intestinal microenvironmental profile that distinguishes IBS patients from healthy subjects, we performed Least Absolute Selection and Shrinkage Operator (LASSO) method with regularization. The caret [4] and glmnet [5] packages in R were used for this purpose (version 3.6.2) [6]. This method models the dependent variables, that can take one of two possible values, i.e. health = 0 or IBS = 1, while reduces the independent variables (i.e. metabolites and bacterial taxa). The variable selection is performed by shrinking the regression coefficients (=slopes) all the way to zero, that leaves the unimportant predictors out of the model and reduces the variance of the original model. With set.seed (100) to generate a sequence of random numbers and assure reproducibility of the results, the combined microbiota and metabolite data frame was randomly split into 80% training dataset and 20% test dataset. As a first step, cv.glmnet function (alpha = 1) in the training set was used to identify the minimum value of lambda (λ_{min}) at the lowest mean-squared error that gives the best model. Then, the Lasso regression was performed by cv.glmnet, glmnet and predict.glmnet functions with the λ_{min} obtained from the previous step against the test dataset [5]. The accuracy of the training dataset in defining a simplified model was determined by comparing the mean of the observed classes with the predicted classes of the test dataset. This process results in identification of the microbial and metabolite variables most strongly associated with the differentiating disease/health groups.

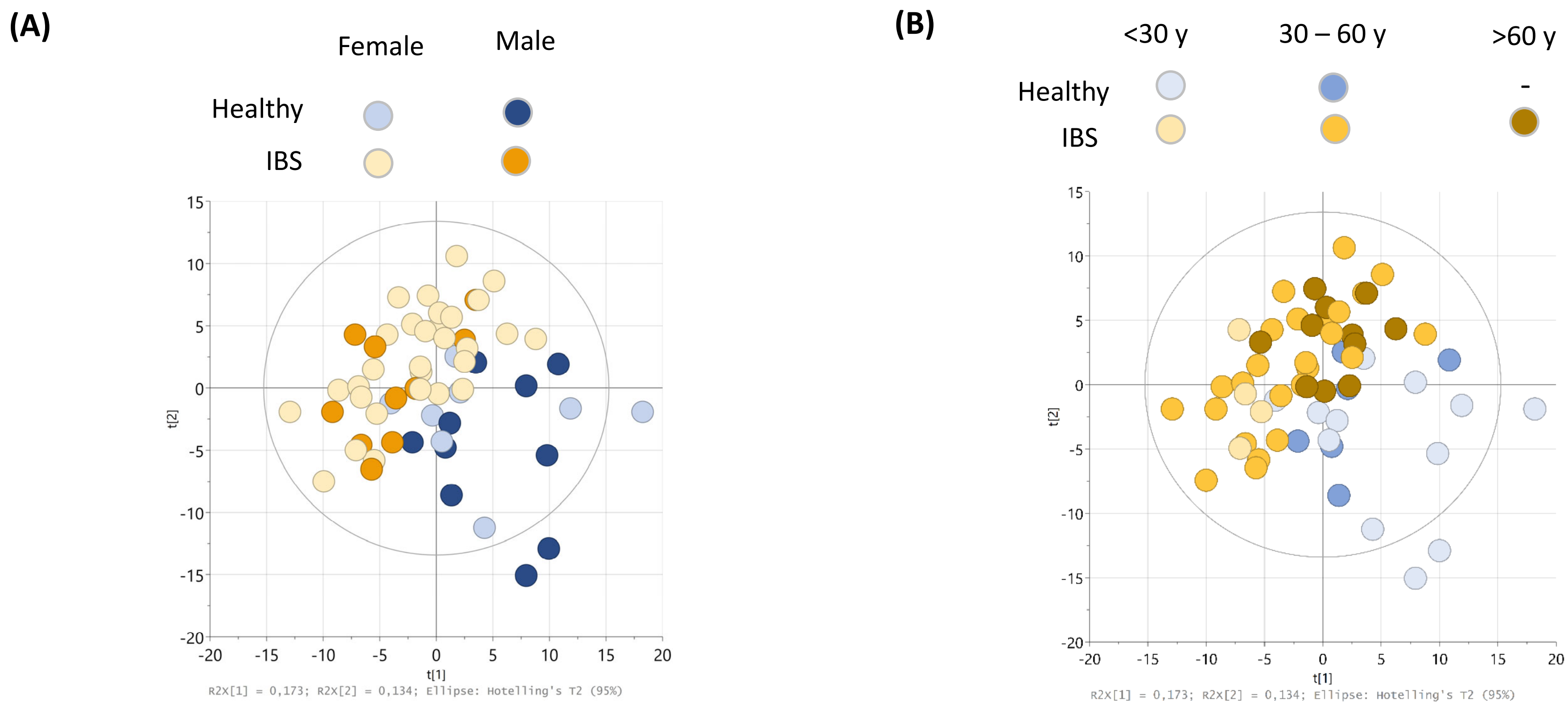

FIGURE S1 Faecal microbiota and metabolite profiles of IBS patients and healthy subjects. (A) Principal component analysis (PCA) score scatter plot based on faecal bacteria and metabolites showing IBS patients (n=40, yellow dots) and healthy subjects (n=18, blue dots) with their identified gender. Light colour represents females while darker colour corresponds to males. (B) PCA score scatter plot based on faecal bacteria and metabolites showing IBS patients and healthy subjects identified by group of age. Age <30 in light, 30 – 60 in intermediate intensity, and >60 in dark yellow (IBS) or blue (healthy), respectively.

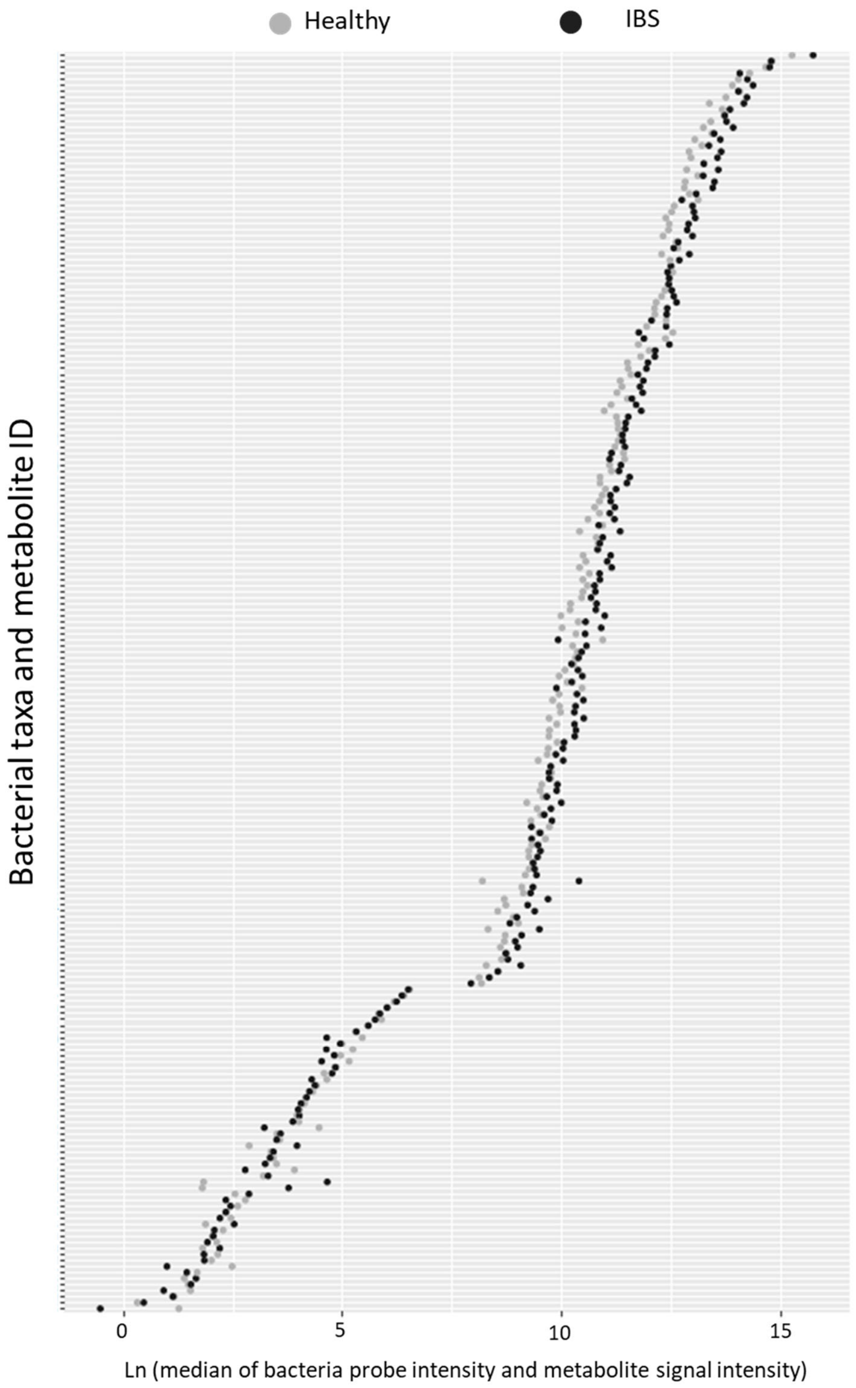

FIGURE S2 Cleveland dot plot representing the distinct combined microbiota and metabolite profile in IBS patients and healthy subjects. The horizontal axis represents bacterial probe intensity (n=54) and metabolite signal intensity (n=155) values and the vertical axis represents the variable IDs. Ln-transformed median values for each X-variable are depicted for the IBS group (black dots) and healthy group (grey dots), respectively.

TABLE S1 Significantly enriched functions predicted by Ingenuity Pathway Analysis (IPA) “Core analysis”

Categories	Biological functions	# Molecules	P value	z-score†
Amino Acid Metabolism, Molecular Transport, Small Molecule Biochemistry	Uptake of amino acids	12	1.04E-07	-3.38
Amino Acid Metabolism, Molecular Transport, Small Molecule Biochemistry	Uptake of L-amino acid	11	1.74E-07	-3.28
Amino Acid Metabolism, Molecular Transport, Small Molecule Biochemistry	Uptake of L-alanine	8	2.60E-08	-2.83
Amino Acid Metabolism, Molecular Transport, Small Molecule Biochemistry	Uptake of glutamine family amino acid	7	3.87E-05	-2.65
Amino Acid Metabolism, Molecular Transport, Small Molecule Biochemistry	Uptake of L-proline	6	3.89E-06	-2.45
Cellular Growth and Proliferation, Organismal Development	Growth of *Yersinia pestis*	7	3.87E-05	2.02
Cell Death and Survival, Organismal Injury and Abnormalities	Apoptosis of epithelial cells	5	3.14E-03	2.18
Cell Death and Survival, Organismal Injury and Abnormalities	Cell death of epithelial cells	8	9.75E-04	2.19
Carbohydrate Metabolism, Molecular Transport, Small Molecule Biochemistry	Uptake of D-glucose	5	1.65E-02	2.21
Free Radical Scavenging	Generation of reactive oxygen species	10	4.03E-03	2.22
Amino Acid Metabolism, Molecular Transport, Small Molecule Biochemistry	Efflux of L-alanine	5	1.49E-05	2.24

Categories	Biological functions	# Molecules	P value	z-score†
Organismal Development	Growth of organism	18	1.09E-10	2.32
Amino Acid Metabolism, Molecular Transport, Small Molecule Biochemistry	Efflux of L-amino acid	8	2.60E-08	2.39
Amino Acid Metabolism, Molecular Transport, Small Molecule Biochemistry	Efflux of neutral amino acid	6	8.95E-07	2.39
Cell Cycle, Hepatic System Development and Function	Entry into S phase of hepatocytes	6	4.20E-09	2.45
Cell Cycle	Interphase	10	5.38E-06	2.56
Cell Cycle	Cell cycle progression	11	1.56E-05	2.58
Cell Cycle	S phase	7	6.34E-07	2.64
Cellular Growth and Proliferation, Organismal Development	Growth of bacteria	15	1.72E-09	2.68
Cell Signaling, Molecular Transport, Vitamin and Mineral Metabolism	Quantity of Ca^{2+}	9	7.56E-03	2.76
Molecular Transport	Quantity of metal	11	3.38E-03	2.92
Molecular Transport	Export of molecule	14	1.52E-06	3.09

Note: Log_2 fold change of mean metabolite signal intensities (n=155) between IBS patients and healthy subjects were analysed with IPA Software to outline the most enriched biological functions. The significant 22 biological functions and the correspondent categories with predicted activation in IBS patients are shown along with the overlapping number of molecules in the datasets (# Molecules), P values, and Activation z-scores. Z-scores < -2 or >2 indicates predicted decrease and increase, respectively.

Abbreviations: IPA, Ingenuity Pathway Analysis; IBS, Irritable bowel syndrome.

†Activation z-score is calculated by the IPA software and predicts whether a specific biological function is increased (positive z-score) or decreased (negative z-score) based on the experimental dataset.

REFERENCES

1. Longstreth, G.F.; Thompson, W.G.; Chey, W.D.; Houghton, L.A.; Mearin, F.; Spiller, R.C. Functional bowel disorders. *Gastroenterology* **2006**, *130*, 1480-1491. doi: 10.1053/j.gastro.2005.11.061.
2. Bjelland, I.; Dahl, A.A.; Haug, T.T.; Neckelmann, D. The validity of the Hospital Anxiety and Depression Scale. An updated literature review. *J. Psychosom. Res.* **2002**, *52*, 69-77. doi: 10.1016/s0022-3999(01)00296-3.
3. Zigmond, A.S.; Snaith, R.P. The hospital anxiety and depression scale. *Acta Psychiatr. Scand.* **1983**, *67*, 361-370. doi: 10.1111/j.1600-0447.1983.tb09716.x
4. Kuhn, M. Building predictive models in R using the caret package. *J. Stat. Softw.* **2008**, *28*, 1-26. doi: 10.18637/jss.v028.i05.
5. Friedman, J.; Hastie, T.; Tibshirani, R. Regularization paths for generalized linear models via coordinate descent. *J. Stat. Softw.* **2010**, *33*, 1-22. doi: 10.18637/jss.v033.i01.
6. R Core Team. R: A language and environment for statistical computing. R Foundation for Statistical Computing: Vienna, Austria, **2020**. http://www.R-project.org/
